# Biliary Tract Cancers: Treatment Updates and Future Directions in the Era of Precision Medicine and Immuno-Oncology

**DOI:** 10.3389/fonc.2021.768009

**Published:** 2021-11-15

**Authors:** Ashish Manne, Edward Woods, Allan Tsung, Arjun Mittra

**Affiliations:** ^1^ Department of Internal Medicine, Division of Medical Oncology at the Arthur G. James Cancer Hospital and Richard J. Solove Research Institute, The Ohio State University Comprehensive Cancer Center, Columbus, OH, United States; ^2^ Department of Internal Medicine, The Ohio State University College of Medicine, Columbus, OH, United States; ^3^ Department of Surgery, Division of Surgical Oncology, The Ohio State University Wexner Medical Center and James Cancer Hospital and Solove Research Institute, Columbus, OH, United States

**Keywords:** biliary tract cancer, cholangiocarcinoma, mutation, methylation, targeted therapy, immunotherapy, biomarker, circulating DNA

## Abstract

The effective management of biliary tract cancers (BTCs) has been hampered by limited options for systemic therapy. In recent years, the focus on precision medicine has made technologies such as next-generation sequencing (NGS) accessible to clinicians to identify targetable mutations in BTCs in tumor tissue (primarily) as well as blood, and to treat them with targeted therapies when possible. It has also expanded our understanding of functional pathways associated with genetic alterations and opened doors for identifying novel targets for treatment. Recent advances in the precision medicine approach allowed us to identify new molecular markers in BTCs, such as epigenetic changes (methylation and histone modification) and non-DNA markers such as messenger RNA, microRNA, and long non-coding RNA. It also made detecting these markers from non-traditional sources such as blood, urine, bile, and cytology (from fine-needle aspiration and biliary brushings) possible. As these tests become more accessible, we can see the integration of different molecular markers from all available sources to aid physicians in diagnosing, assessing prognosis, predicting tumor response, and screening BTCs. Currently, there are a handful of approved targeted therapies and only one class of immunotherapy agents (immune checkpoint inhibitors or ICIs) to treat BTCs. Early success with new targets, vascular endothelial growth factor receptor (VEGFR), HER2, protein kinase receptor, and Dickkopf-1 (DKK1); new drugs for known targets, fibroblast growth factor receptors (FGFRs) such as futabatinib, derazantinib, and erdafitinib; and ICIs such as durvalumab and tremelimumab is encouraging. Novel immunotherapy agents such as bispecific antibodies (bintrafusp alfa), arginase inhibitors, vaccines, and cellular therapy (chimeric antigen receptor—T cell or CAR-T, natural killer cells, tumor-infiltrating lymphocytes) have the potential to improve outcomes of BTCs in the coming years.

## Introduction

Biliary tract cancers (BTCs) are a heterogeneous group of aggressive malignancies that arise from the epithelium of the biliary tract, which includes the bile ducts and gallbladder (1). Cholangiocarcinoma (CCA) includes tumors arising from the bile ducts and are classified anatomically as either intrahepatic and extrahepatic depending on the part of the biliary tract they originated from (2). BTCs are rare and aggressive tumors with a 5-year survival rate for metastatic disease being only 2% (3). In 2017, around 200,000 BTC cases were reported worldwide (4). The incidence and mortality increased by 76% and 65%, respectively, in the last quarter-century (1997–2017). It is difficult to estimate the incidence and mortality of BTC in the United States as epidemiologic data on intrahepatic cholangiocarcinoma (IHC) and hepatocellular cancers (HCC) are reported together, while extrahepatic cholangiocarcinoma (EHC) and gallbladder cancers (GBC) are reported as one group (5). In 2021, it is estimated that there will be approximately 42,000 new cases and 30,000 deaths from HCC & IHC. For EHC & GBC this estimate approximately 12,000 and 4,000, respectively (5). Peri-hilar CCAs are usually classified under EHC.

While surgery is the only curative treatment, unfortunately, the majority of patients with BTC (60%–70%) present with advanced or metastatic disease, and therefore, palliative locoregional and systemic therapy are the only options for treatment (6). Based on the results of the ABC-02 trial, the combination of gemcitabine and cisplatin has become the standard first-line treatment for advanced biliary tract tumors demonstrating a median overall survival of 11.7 months (7). For the modest proportion of patients who go on to receive second-line chemotherapy, the guidelines are less clear about the appropriate therapy for patients who have progressed on Gem-cis and still maintain adequate functional and lab status to tolerate more therapy. Recently, the phase-3 ABC-06 trial showed a survival advantage of FOLFOX over active symptom control (ASC) for patients who had received Gem-cis in the first line (8). A systematic review of second-line therapies in biliary cancers demonstrated a mean PFS of 3.2 months and a mean overall survival (OS) of 7.2 months (9). However, for patients whose tumors harbor targetable mutations, targeted therapy is preferred over chemotherapy in the second line. These include pemigatinib and infigratinib for fusions or mutations in fibroblast growth factor receptor-2 (FGFR2), larotrectinib and entrectinib for neurotrophic tropomyosin receptor kinase (NTRK) fusions, and ivosidenib for isocitrate dehydrogenase 1 (IDH1) (10–14). Pembrolizumab, an immune checkpoint inhibitor (ICI), is recommended for patients with microsatellite instability-high (MSI-H) (15). Unfortunately, only a small proportion of patients have tumors harboring these specific mutations. There is, therefore, an urgent need to expand the arsenal of therapeutic options to treat BTCs and identify biomarkers with reliable prognostic and predictive value. It is imperative that precision medicine strategies should go beyond only somatic mutations to help realize this goal.

## Precision Medicine in Biliary Tract Cancer: Current State and Future Directions

Precision medicine refers to tailoring an approach specific to an individual at the molecular level (16). It gained a greater role in oncology over the last two decades, partly driven by easier access to next-generation sequencing (NGS)-based comprehensive genomic profiling (CGP) that enables detection of alterations in the genome, including base-pair substitutions or single nucleotide polymorphisms (SNP), copy number variations (CNV), insertions/deletions, and rearrangements (17). Precision medicine enables us to understand the genomic landscape of BTCs that in turn shed light on the pathways responsible for the malignant transformation and drug resistance and ultimately in effective drug discovery. Moreover, growing evidence shows that IHC, EHC, and GBC have noteworthy differences in their respective genomic landscape (as discussed below).

**Figure 1:**
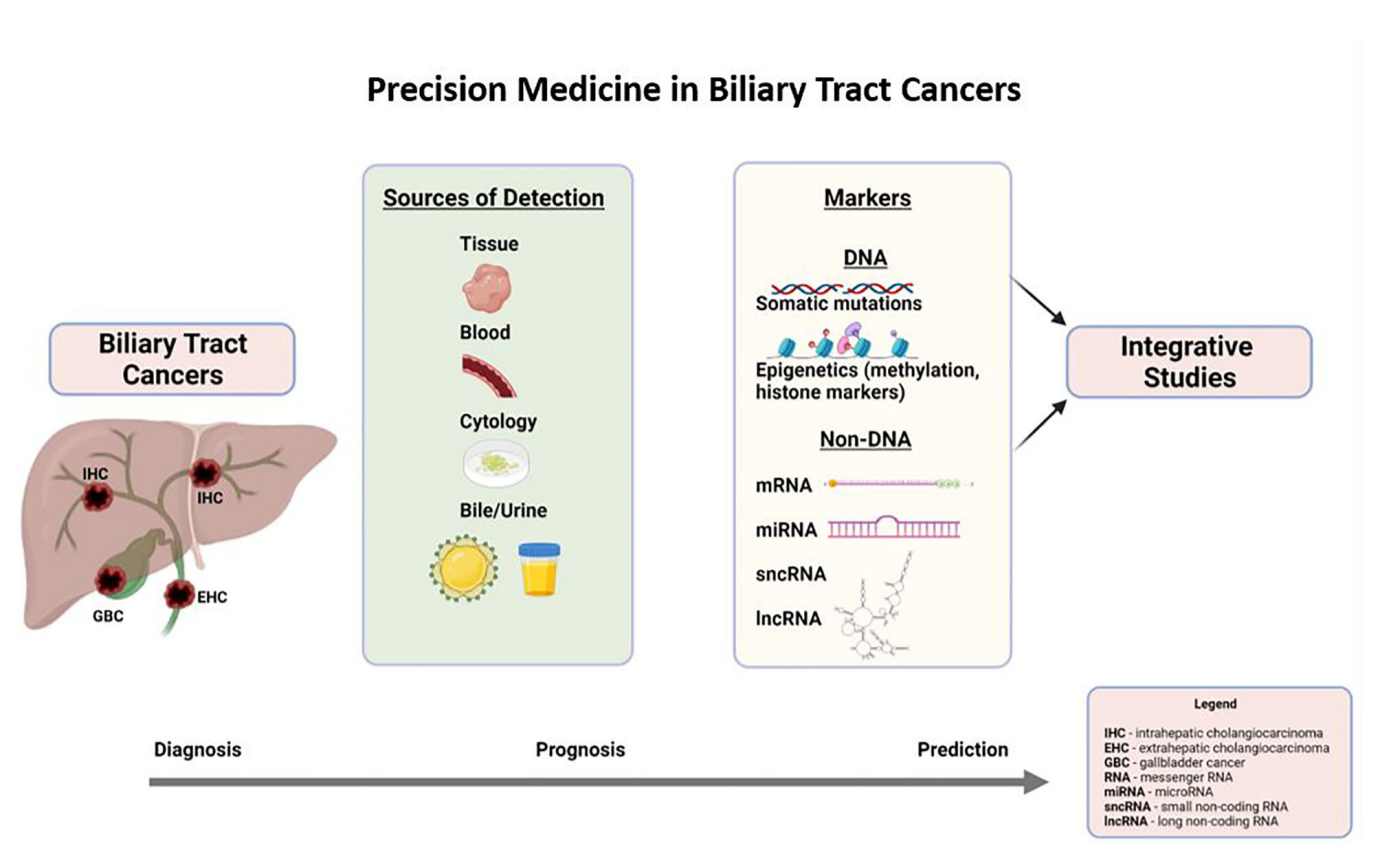
Precision medicine in biliary tract cancers.

Precision medicine is synonymous with mutational profiling in tumor tissues, precisely for targetable mutations in current practice. In the last 5 years, there has been remarkable progress in identifying other DNA molecular markers such as epigenetic markers and non-DNA molecular markers such as RNA markers (coding and non-coding), metabolites, and protein markers in the tumor tissues. Moreover, the fruits of precision medicine were extended to other sources of tumor genetic material (DNA and RNA) such as blood, bile, urine, and cytology (biliary brushings), which is exciting. This part of the discussion will focus on DNA markers, including somatic mutations, associated signaling pathways, and epigenetic markers (DNA-methylation markers) in tumor tissue, blood, bile, and cytology (as illustrated in **Figure 1**). Non-DNA markers, such as messenger RNA and non-coding RNA, will be discussed briefly. Prognostic and predictive markers are presented in each section if the evidence is available.

### Somatic Mutations and Functional Pathways in Biliary Tract Cancer in Tumor Tissue

The most frequently mutated genes in the tumor tissue of BTCs are *TP53*, *KRAS*, *CDKN2A/B*, and *SMAD4* (18–24). The same studies reported low prevalence (usually <5%) of targetable mutations such as *IDH1/2*, *FGFR2*, *BRAF*, *PIK3CA*, and *NTRK*. It is difficult to estimate the frequency of each genetic alteration for the entire group (EHC, IHC, and GBC) as the study populations were different in these studies. Among BTCs, there are some noticeable differences in the detected mutations, which are summarized in **Table 1** (22, 25–34).

**Table 1:** Prominent differences in mutated genes among the BTCs.

Specific BTC	Most likely exclusive	Most frequent	Least frequent
Intrahepatic cholangiocarcinoma	Present—*IDH1*/2	*BAP1*, *CDK2NA*, *ARD1A*, *FGFR1–3*, *MET*	*TP53*, *PIKCA*, *HER2*
Extrahepatic cholangiocarcinoma	Absent—*FGFR1*–3, *MET EGFR*	*KRAS* ^b^	*CDK2NA*/B, *ARD1A* ^a^
Gall bladder carcinoma	No exclusive mutations	*TP53*, *PIK3CA*, *HER2*, *BRAF*, *EGFR*	*BAP1*

a Mutations less frequent in IHC compared with EHC along with KRAS, HER2, and SMAD4.

b Mutations more frequent in IHC compared with EHC along with PBRM1.

Targetable mutations with approved therapies (*FGFR2* and *IDH1/2*) are more common in IHC, and *IDH1/2* mutations rarely occur in EHC or GBCs (32, 33). Eight genes identified as potential drivers for IHC are *TP53*, *KRAS*, *IDH1*, *PTEN*, *ARID1A*, *EPPK1*, *ECE2*, and *FYN* (30). In multifocal metastatic IHC, the SNPs and CNVs in the primary are often concordant with the intrahepatic metastasis (same segment and <2 cm from the primary) and satellite lesions (different segment and >2 cm from the primary) (35). Clinically, this may indicate that multifocal IHCs originate from common progenitor cells and can be considered for surgical resection.

EHCs rarely have targetable mutations and are more likely than other BTCs to harbor a *KRAS* mutation. Precursor lesions, intraductal tubular papillary neoplasm (ITPN), predominantly originate in intrahepatic ducts and have very few mutations (36). Intraductal papillary neoplasm of the bile duct (IPNB) is usually localized in extrahepatic ducts and has mutational profiles similar to EHC. Overall, the precursor lesions and invasive CCA have overlapping mutations with few exceptions: *ROBO2* mutations exist only in invasive CCA, and *CTNNB1* are identified in ITPN and IPNB (36). *TP53* is the most common mutation in GBC, while *PIK3CA* is the least prevalent (32, 33). *HER2* alterations are common in GBC compared with CCAs. CNVs in *CDKN2A*, *TP53*, *MDM2* proto-oncogene, and *CCD1* genes and *HER2* amplifications increased with the development GBC from its precursor lesions (gallstones, low-grade/high-grade dysplasia) (37). These distinct mutational profiles among BTCs can help localize the origin of the tumor and tailor therapy for individual patients.

Mutation detection in tumor tissue or blood helps identify the cell signaling pathways that play a pivotal role in carcinogenesis, drug resistance, and prognosis. Tools such as Ingenuity Pathway Analysis (IPA) were used in previous studies to correlate the genomic variations with specific signaling pathways (27). The major pathways and associated gene alterations in BTCs are as follows: i) FGF pathway with FGFR mutations; ii) mTOR with mutations such as *FBXW7*, *PIK3CA*, *PTEN*, *NF1*, *NF2*, *PIK3R1*, *STK11*, *TSC1*, and *TSC2*; iii) MAP/ERK pathway with *KRAS*, *MYC*, *BRAF*, *EGFR*, *MAP2K1*, *MAP3K1*, and *NRAS*; iv) DNA damage repair (DDR) pathway with *MSH6*, *BRCA1*, *BRCA2*, *BAP1*, *ATM*, *MLH1*, and *MSH2*; and v) chromatin remodeling (CR) modification pathway with *BAP1*, *ARID1A*, and *PBRM1* (27).

About 19% of BTCs have DDR gene alteration mutations and usually co-exist with CR alterations such as *ARID1A* and *PBRM1* mutations (38). These tumors tend to have a high tumor mutational burden (TMB) and a worse prognosis (38, 39). cAMP-dependent signaling activation is another pathway common for all three types of BTCs (25). Nepal et al. classified IHCs based on the three common mutations, *IDH*, *KRAS*, and *TP53*, or undetermined, and showed their potential predictive value in cell lines. *IDH*-mutated IHCs are rich in metabolic pathways such as glutathione metabolism and the citrate cycle and respond to metabolic modulators such as IDH1 inhibitors. *KRAS*-mutated IHCs are rich in immune-related pathways and actin cytoskeleton rearrangement and may benefit from microtubule modulators or immunotherapy. *TP53*-mutated tumors are rich in cell cycle dysregulation (MAPK, WNT, and p53 signaling) and may benefit from topoisomerase inhibitors. In IHC without *IDH*, *TP53*, and *KRAS* mutations, the mTOR pathway is predominant and may benefit from mTOR inhibitors. The relation between IDH-mutated tumors and metabolic enzymes was observed in other studies too, but rigorous preclinical and clinical studies are needed before this classification can be used in clinical practice (32).

Mutational profiling can also aid in identifying the etiology of BTCs. Fluke-positive (*Opistharchis viverrine* and *Clonorchis sinensis* related) tumors predominantly have *KRAS*, *TP53*, *KMT2C* (*MLL3*), *ROBO2*, *RNF43*, *PEG3*, *GNAS*, *SMAD4*, *BRCA1*/2, and *HER2* compared with fluke-negative tumors (40–42). *BAP1*, *IDH1*/2, and *FGFR* mutations are frequent in the latter group (41, 42). In IHCs, HBsAg-seropositive patients most likely have *TP53* mutations, and *KRAS* mutations are common in HBsAg-seronegative patients (30). IDH alteration-positive IHCs usually do not have any underlying risk factors such as infections, bile duct cysts, alcohol/tobacco, or Thorotrast exposure (33).

### Prognostic and Predictive Value of Somatic Mutations in Biliary Tract Cancer

The somatic mutations with potential prognostic value are summarized in **Table 2** (20, 22, 26–28, 32, 42–45). Detection of *FGFR* and/or *IDH1* in IHC and *PBRM1* and/or *BAP1* in EHC is associated with a good outcome. Alternatively, *PIK3CA* in EHC and specific mutations in IHC, such as *TP53*, *KRAS*, *CDK2NA/B*, *EGFR*, and *PBRM1*, are bad prognostic markers. Mutations with prognostic value are not well established for GBCs. CCAs with mismatch repair (MMR) deficiency and low mesothelin levels have poorer outcomes compared with those with MMR proficient and high mesothelin expression (median OS: 14.5 *vs.* 30.0 months, *p* = 0.05) (46). In the same study, CCA with MMR deficiency alone had a trend toward the worst prognosis (median overall survival or OS: 19.2 *vs.* 28.1 months, *p* = 0.07). In IHC, Zhu et al. have demonstrated an interesting relation between mutations and pathological features: a) *IDH1* alone or *IDH/IDH2* is associated with bilobar invasion of the tumor; b) *KRAS* alone with positive margins (R1) and direct invasion of surrounding organs and *KRAS*, *NRAS*, or *BRAF* with R1; and c) *NRAS* with intrahepatic metastasis (47).

**Table 2:** Somatic mutations and their prognostic value in biliary tract cancers.

Tumor group	Worse	Better	No effect
Biliary tract cancers	*ARID1A* *KRAS* ^a^ *Del at 7q22.1* *High TMB*	*FGFR2* *PRB1A*	*CDKN2A*, *CDKN2B*, *IDH*, *PIK3CA*, *MYC* alteration
Intrahepatic cholangiocarcinoma	*TP53* *KRAS* *CDK2NA*/B *EGFR* *BAP1* ^a^ *ARID1A* ^a^ *PBRM1* *BRCA1*/2	*FGFR* (point mutations and translocations) *IDH1^b^*	*CDK2NA* *BRAF* *PTEN* *HER2*
Extrahepatic cholangiocarcinoma	*PBRM1* *BAP1* ^c^	*PIK3CA*	*TP53* *KRAS* *CDK2NB* *A1RD1* *PBRM1* *SMAD4*
Gallbladder cancers	None identified	None identified	*TP53* *KRAS* *CDK2NA/B* *ARID1A* *IDH1* *PIK3CA* *SMAD4* *MYC*

TMB, tumor mutational burden.

a No effect in one study and bad prognosis in others.

b No effect in some studies and good prognosis in others.

c PFS is different but not OS.

Studies have shown that patients with targetable mutations have better response and survival advantages if treated with the appropriate targeted therapy (20, 21). Therefore, it is crucial to attempt to identify targetable mutations when possible. For patients with no targetable mutations, there are no established mutations with predictive value. EHCs with overexpression of programmed cell death protein 1 (PD-1)/programmed death-ligand 1 (PD-L1) and higher lymphocyte infiltration respond better to ICIs (48). There are no mutations that can predict response in patients receiving chemotherapy either. With limited treatment options and poor outcomes in BTCs, this area needs further study. One area where this would be used is in the neoadjuvant setting where the lack of treatment response to chemotherapy could be identified upfront, and alternate therapy such as targeted agents or immunotherapy could be used instead (49).

### DNA-Methylation Markers and Integrative Approach in Biliary Tract Cancer

Epigenetic changes are modifications in the genome not involving the nucleotide sequence (50). They can be a) DNA hypo- or hypermethylation; b) histone modification including methylation, phosphorylation, acetylation, and SUMOylation; c) CR; and d) RNA-associated silencing. In this section, the focus will be on DNA-methylation markers. Comparing the methylation profiles of malignant *vs.* normal or precancerous tissues can provide insight into pathways of malignant transformation and cells of origin in BTCs (36). Integrating methylation profiles with clinical and/or mutational profiles has prognostic and predictive value (28, 29, 42, 51).

In CCAs, frequently methylated genes are *APC*, *DAPK*, *E-cadherin*/*CDH1*, *GSTP*, *RASSF1A*, *hMHL1*, *MGMT*, *p15^INK4b^*, *p16^INK4a^*, *p^14ARF^*, *p73*, *14-3-3 sigma*, *SOCS*-3, *EGFR*, and *RAR-β* (52, 53). *RASSF1A*, *HOXA1*, *HPP1*, *CDH1*, and *NEUROG1* are predominantly methylated genes in EHC, while *CHFR*, *GSTP1*, *IGF2*, *MGMT*, *MINT31*, *p14*, and *RBP1* are predominant in IHC (52, 54). MGMT promoter methylation level is high, and protein expression is low in IHC compared with normal tissues (55). MGMT inhibition promotes cell proliferation *via* p21, P27, and Cyclin E. Low-expression MGMT in tumor tissues is associated with worse clinicopathological features and outcomes. Similarly, promoter methylation of APC, p16, and TIMP3 have a good prognostic value in IHC (56). EHCs with lymph node metastasis have higher CpG island loci and hypermethylation of *T1G1* gene (54). A data mining study in an extensive database identified genes associated with methylation pathways in GBC (*FGA*, *F2*, *HAO1*, *CFH*, *PIPOX*, *ITIH4*, *GNMT*, *MAT1A*, *MTHFD1*, *HPX*, *CTH*, *EPHX2*, *HSD17B6*, *AKR1C4*, *CFHR3*, *ENNP1*, and *NAT2*) (57). Among them, the methylated genes were *FGA*, *CFH*, *F2*, *HPX*, and *PIPOX*. When validated in the clinical samples, *FGA*, *CFH*, *F2*, *HPX*, and *PIPOX* were high compared with controlled tissues but not significantly different. Multiple studies identified numerous genes methylated in GBC, and a set of genes consistently represented in these studies is as follows: *APC*, *SHP1*, *3-OST-2*, *FHIT*, *p16*, *SEMA3B*, and *CDH13* (58).

Integration of the genomic (somatic mutations, CNVs, and gene expression) and epigenomic data (methylation) has been attempted to better understand the disease processes. In such studies, the tumors are classified into groups, or clusters, to identify high-risk populations. In one such study, IHCs were divided into four clusters using the iClusterPlus platform (42). Cluster 1 mainly had fluke-positive IHCs rich in *TP53*, *ARID1A*, *BRCA1*/2, and *H3K27me3* promoter mutations; had high expression of *HER2*, *AKT1*, and *EZH2*; and low expression of *TET2* and CpG island hypermethylation. Clusters 2 and 3 had a mix of fluke-positive and fluke-negative tumors. Cluster 2 tumors were rich in *TP53* mutations and had high expression of *CTNNB1*, *WNT5B*, and *AKT1*. Cluster 3 was rich in immune-related pathways and had the highest CNVs. Cluster 4 had tumors rich in *BAP1*, *IDH1*/2, and *FGFR* mutations; high *FGFR1–4* gene expression; and CpG shore island hypermethylation. Cluster 4 had a better prognosis over clusters 1–3. Qui et al. divided BTCs into six clusters based on the degree of methylation (51). The cluster with a high methylation rate had the highest CNV and a worse prognosis. Lower methylation rate tumors had higher BCR/TCR diversity, immune cell infiltration, and PD-L1 and cytotoxic T-lymphocyte-associated protein 4 (CTLA-4) mRNA expression and, hence, potentially respond to ICI better. Interestingly, hypermethylated genes participate in DNA-binding transcription activity, and hypomethylated genes are involved in transmembrane receptor and ion binding.

In another study, the authors integrated gene expression data, signaling pathways, chromosomal abnormalities, mutations, and poor prognosis signatures derived from previous studies in HCC and tyrosine kinase inhibitors in CCA (28, 59–61). They divided the IHCs into two classes: “proliferative” and “inflammatory”. The proliferative class accounted for 62% of the study population. These tumors had *KRAS* and *BRAF* mutations; more oncogenic pathways such as RAS/MAPK and MET; poor prognostic signatures; specific CNVs, amplifications at 11q13.2, deletions at 14q22.1; and moderate to poor differentiation with intraneural invasion on histology and poor survival. In the inflammatory class, tumors had activated inflammatory signaling pathways, overexpression of cytokines (interleukin or IL-3, IL-4, IL-6, IL-10, IL-17A, and CCL19), and STAT 3 activation; no poor prognostic signatures, KRAS or BRAF mutations; and well-differentiated tumors with favorable features and good survival.

IDH-mutant CCAs have a distinct molecular profile, according to a study that integrated genomic (somatic mutations and CNV) and epigenomic data (DNA-methylation) with mRNA expression (29). They have higher mitochondrial DNA copy numbers, low expression of chromatin modifiers, and elevated expression of mitochondrial genes compared with IDH-WT tumors. Comparing 103 IHCs with matched controls, Zhou et al. identified three pathways (transforming growth factor-b/Smad signaling pathway along with known Ras/phosphatidylinositol-4,5-bisphosphate 3-kinase signaling and p53/cell cycle signaling) along with genes involved in epigenetic regulation and oxidative phosphorylation more frequent in IHCs (30). Using samples from patients in Chile (which has the highest incidence of GBC), Brägelmann et al. were able to identify methylation changes during the progression from gallstone disease to dysplasia and then to GBC (37). They identified stages of progression through a sequence of early (gallstone disease and low-grade dysplasia), intermediate (high-grade dysplasia), and late (GBC) stages. In particular, methylation of genes involved in WNT signaling, Hedgehog signaling, and tumor suppression increased with tumor grade. CNVs also increased along with tumor grade (as mentioned above).

### Somatic Mutations in Blood, Bile, and Cytology Specimens in Biliary Tract Cancer

Circulating cell-free DNA (cfDNA) usually refers to nucleic acids (fragments of DNA) detected in the peripheral blood released secondary to apoptosis or necrosis (62, 63). In cancer patients, a significant proportion of cfDNA comes from normal cells of the body, a small part related to tumors, coming from primary tumors, metastatic sites, or circulating tumor cells (CTC), and is called circulating tumor-DNA (ctDNA) (64). In the last 5 years, liquid biopsies (detecting mutations in cfDNA) have gained popularity in oncology practice as they may be the only source for tumor DNA in certain cancers such as BTCs, where getting adequate tissue for sequencing is difficult. Among BTCs, acquiring tissue for EHCs is more difficult than for IHC and GBCs. In BTC, limited studies in this area indicate a high mutation detection rate (74%–85%) regardless of whether patients are treatment naive or on treatment at the time of sample collection and have reliable concordance with mutations in the tumor tissue (50%–74%) (21, 65–67). Concordance in the matched samples (blood and tissue from the same patient) depends on the source of the tissue (higher with metastatic *vs.* primary site) and also the tumor type [higher in IHC (92%) compared with EHC or GBC] (67).

The cfDNA has a dependable sensitivity and specificity in diagnosing BTC (68, 69). The frequency of the detected mutations in cfDNA in BTC patients is the same as those detected in the tumor tissue (21, 67). Due to limited studies, it is hard to conclusively identify cfDNA mutational profile differences between IHC, EHC, and GBCs. In one study with 69% IHCs, 18% EHC, and 13% GBC, *FGFR* and *ARID1A* were the most prevalent alteration in IHC and EHC, respectively. *CDK6*, *APC*, and *SMAD4* alterations were common in GBCs. *TP53* and *KRAS* were the most prevalent in all three groups (70). BTCs in younger patients (<50 years) have more *FGFR2*, *PIK3CA*, *MET*, and *BRAF* mutations compared with older patients (≥50 years) (70). *TP53* (67% *vs.* 35%, *p* = 0.6) was predominant in the older population.

Detection of clinically relevant and targetable mutations for approved therapies is better in ctDNA than tissue, and it ranges between 30% and 40% in advanced CCAs (65, 71). In a study with 71 BTCs with detectable ctDNA, 75% of the patients had targetable mutations (both on- and off-label for BTC), indicating that this will be a valuable tool in the future (21). Variant allelic frequency (VAF) represents the percentage of mutant reads divided by the total number of reads coverage at a specific genomic position (72). VAF in ctDNA at the baseline correlates well with the tumor burden in CCA but does not have any predictive or prognostic value (65, 67). cfDNA integrity (ratio of ALU247 and ALU115) had a reliable diagnostic and prognostic value (unfavorable clinicopathological features) in GBC (68). Short segments of DNA originally characterized by *Arthrobacter luteus* (Alu) restriction endonucleases are ALU units (69).

CTC detection is low in CCA, especially in early-stage (stages I and II) tumors, and detection rates vary with the thresholds for positive tests (CTC ≥ 2 *vs.* CTC ≥ 5). In a study reported in 2016, detection rates for the threshold of ≥2 and ≥5 were 17% and 9%, respectively, and most of them were advanced-stage cancers (73). Both the thresholds had significant prognostic value in the entire group. In subgroup analyses, both maintained prognostic value in metastatic CCA and EHC. A threshold of ≥5, in addition, had prognostic value in the IHC group and trended toward significance in non-metastatic CCAs. Li et al. proposed the concept of ctDNA fingerprinting in eight tumor types, including CCA (74). The authors began with whole exosome sequencing (WES) in patients before the treatment (surgery or systemic therapy or locoregional therapy) appropriate to the disease. Then, a patient-specific panel with high-frequency clonal population clusters was designed and followed in the patients after treatment. Two entities followed in subsequent tests were ctDNA content fraction (CCF) and the fold change in CCF. The CCF is higher, and the fold change increased in patients who progress compared with patients with stable disease or response. In patients receiving selective internal radiation, a reduction in the CNVs was observed in patients with IHC (75). Platinum-based therapy showed a potential benefit in BTCs with DDR gene mutations in a small study (65). The benefit was seen in partial response rate, radiological response, and increased overall survival.

In one of the earlier studies that proved the feasibility of detecting DNA-methylation markers in CCA, a four-gene panel with *HOXA1*, *PKRCB*, *CYP26C1*, and *PTGDR* was proposed to differentiate CCA from healthy controls with sensitivity/specificity of 83%/93% (76). A couple of years later, higher cfDNA methylation levels of *OPCML* and *HOXA9* genes, individually or together, also proved to have a good diagnostic value in distinguishing CCA from benign biliary diseases (77). The sensitivity/specificity of a combined marker is 62%/100%. Non-specific methylation levels in cfDNA do not have good diagnostic utility in GBC, with sensitivity/specificity around 55%/50% (68). We need robust studies to take the methylation markers in cfDNA to clinical practice.

Examining cytology specimens derived from endoscopic retrograde cholangiopancreatography (ERCP) to identify malignant cells is the standard test to diagnose malignancy. The addition of fluorescence *in situ* hybridization (FISH) and mutational profile testing to cytology helps to diagnose BTCs in patients who present with biliary strictures (78–81). Mutational profiling with *KRAS* and other prominent tumor-suppressor genes alone can increase the sensitivity by 56% and diagnostic yield to 100% when done in conjunction with cytology (82).

DNA-methylation markers in the biliary brushings can also be a tool for diagnosing CCA, and many studies reported using four to six gene panels for this purpose. Andersen et al. suggested that a four-gene panel, namely, *CDO1*, *CNRIP1*, *SEPT9*, and *VIM*, detected in 45%–77% of the samples had a sensitivity and specificity of 85% and 98%, respectively (83). Prachayakul et al. reported using the methylation index (MI) of two genes, *HOXA1* and *NEUROG1* (84). Each has better sensitivity and accuracy than cytology and CA 19-9 in differentiating CCA from benign diseases. The methylation markers were positive even in patients with negative cytology and normal CA 19-9 levels. The combined sensitivity/accuracy of MI, cytology, and CA 19-9 was 97%/91%. Yang et al. reported good sensitivity and specificity of methylated EMX1 for differentiating EHC from primary sclerosing cholangitis (PSC) and non-PSC controls (85). In the same study, *HOXA1* had a sensitivity of 100% differentiating CCA from non-PSC controls, but not in PSC controls. Parsi et al. showed that 80% (*N* = 10) of NTCs have positive methylation for one of three genes, *CCND2*, *NPTX2*, and *TFPI2* (86).

The ctDNA isolated from the bile using the techniques employed for plasma cfDNA (NGS) has shown some encouraging results (87, 88). In bile, mutations can be detected in 60%–75% of BTC patients, with specificity close to 100%. Concordance rates between bile ctDNA and tissue DNA are higher than plasma cfDNA (88%). *p^16INK4a^* is a tumor-suppressor gene frequently associated with many cancers, including CCA (89). In bile samples, promoter methylation of *p^16INK4a^* is positive in over 50% of CCA compared with just 6% in benign diseases (cholelithiasis) and normal bile ducts (90). Concordance with the tissue samples for *p^16INK4a^* promoter methylation was as high as 86%. Just as with mutation profiling of biliary brushings, few panels for methylation markers in bile have been reported in the literature. In EHC, methylation of the five-gene panel with *CCND2*, *CDH13*, *GRIN2B*, *RUNX3*, and *TWIST1* has a better sensitivity than cytology (83% with methylation markers *vs.* 46% in cytology) (91). Similarly, Zhang et al. proposed a six-gene panel (*DKK3*, *p16*, *SFRP2*, *DKK2*, *NPTX2*, and *ppENK*) to diagnose pancreatobilary cancers with sensitivity/specificity/accuracy of 77.27%/77.78%/77.50% (92). In summary, expanding precision medicine to bile and cytology brushings can improve diagnostic testing and help identify better prognostic and predictive biomarkers.

### Non-DNA Molecular Markers in BTC Tissues

Non-DNA molecular markers include messenger RNA (mRNA), non-coding RNA (ncRNA), proteins, and metabolites. mRNA and ncRNA are within the scope of precision medicine. The ncRNA refers to RNA that is not translated into protein and can be detected in the blood and is being extensively studied in tumors for diagnostic and prognostic purposes (93, 94). They can be classified bases on their size into a) long non-coding RNAs or lcRNAs that are more than 200 bases; and b) small non-coding RNAs or sncRNAs that have up to 200 bases which are further classified into small nucleolar RNAs (snoRNAs), small nuclear RNAs (snRNAs), Piwi-RNAs (piRNAs), and microRNA or miR. Extracellular vesicles are lipid membrane-bound spheres released from cells into body fluids (blood, saliva, bile, and urine) comprising materials shed from the cells, including proteins, nucleic acids, and metabolites (95, 96). They are a good source for molecular markers in the blood. They are of three kinds: microvesicles, exosomes, and apoptotic bodies (97, 98).

Among the RNA molecular markers, miR is the preferred biomarker among all the ncRNAs in oncology as it has a proven role in pathways implicated in malignant transformation and can be detected/measured easily in blood, bile, and other body fluids; it is a more stable nucleic acid making it more reliable (94, 99–101). Even though it is still in the experimental stage, the current landscape of prominent non-DNA markers in blood and urine is summarized in **Table 3** (44, 102–128).

**Table 3:** Non-DNA markers in biliary tract cancers.

Source	Non-DNA marker
Tissue	miR-22, -125a/b, -127, -199a, -376a/c, -142-3p, -25, -15a/b, -193, -17-5p, -374, -106a/b, -224, -130b, -19a, -331, -324-5p, -20, 17-3p, -223, -15b, -103, -98, -204, -338, -198, -302d, -328, -337, -302b, -184, -320, -371, -185, -222, -214, -373, -145, -200c, let-7a, let-7b, -21, -135b, -122, -27a, -29a, -429, -24, -203, -29b, -20a/-20b, -93, -30e, -30b, -151-3p, -10a, -181a, -96, -663b, -103, -221, -107, -424, -340, -451, -145, -99a, -630, let-7c, -144, -100, -139-5p, -337-3p, -1, -126, -376c, -517c+-519a, -520e, -30c, -96, -30b, -100, -145 lncRNA PANDAR 141, AFAP1‐AS1 140, CCAT-1, NEAT-1, MALT-1, CPS1-IT1 Prognostic value: miR-192, -675-5p, -652-3p, -338-3p, -126, -21, 192, -21, -214, -151-3p, -373
Blood	In circulating-free RNA: mRNA^H/PSC/UC^: CMIP, GAD1, NME1, CSD1, NME1, CDS1, CK1B, CKS1B; miR-21^HI/b^, -221^H/b^, -194 with miR483-5p^H/b^, -222 with miR483-5p^PSC^, -122^H^, -192h^I/Liverfluke^, 26a^H^, -150^H/I^, -106A^HL^, -26a^PSC^, -122^PSC^, -1281^PSC^, -126^PSC^, -30b^PSC^ In EV: miR-604^H^, -1224^H^, -200c-3p, -96-5p, -151a-5p, -191-5p, -4732-3p, -551B^H/PSC/UC^, -200a/c-3p: lcRNA—LOC100134868, LOC643955. PTTg3P Prognostic value: seven mRNA signatures, namely, CD36, GGCX, UBASH3B, DBN1, PTTG1, CCNA2, and SPATS2. In resected tumors, postop decline in total miR level (by 20), miR-106a, -192, -26a, -150. miR-200a/c-3p (in EV)
Bile	Circulating-free miR9, -145, -105, -147b,let-7f-2,let-7i,-302c,199a-3p, -222^a^, -942, *-640* ^PSC^, -412^PSC^, -1537^PSC^, -3189^PSC^, -30d-5p^Benign^, -92a-3p^Benign^; higher methylation rates of miRNA-1247 and -200a In EV: miR-191, -486-3p, -1274b, -16, -484
Urine	Circulating-free mRNA: UBE2C, SERPINB1; miRNA 21 and 192^H^ In EV: miR-483; snRNA—RNU11: miscRNA—LOC257358: vtRNA—RNA1-1

H, compared to healthy; PSC, compared to primary sclerosing cholangitis; UC, ulcerative colitis; I, exclusive for IHC; B, include all BTCs; Benign, compared to benign; mRNA, messenger RNA; miR, micro RNA; lncRNA, long non-coding RNA; EV, extra vesicles; miscRNA, miscellaneous RNA; vtRNA, vault RNAs; snRNA, small nuclear RNA.

a miR122 is lower compared with PSC and higher in healthy.

In summary, NGS of the tumor tissue (when available) is widely used to identify the targetable mutations in the current clinical practice for managing BTCs. There is a strong clinical need to develop novel biomarkers. On the clinical side, we may see the expansion of precision medicine in three different directions soon: firstly, identifying more reliable DNA-molecular markers such as DNA-methylation markers and non-DNA markers such as mRNA, miRNA, and lncRNA; secondly, making detection of the molecular markers in non-invasive sources of genetic material such as blood (ctDNA and EV), bile, and urine feasible with acceptable reliability and accessible in day-to-day clinical practice; and finally, integrating different molecular makers from different sources and stratifying in high prognostic and predictive value. Non-invasive sources will also help in monitoring the treatment response. Novel techniques to detect ctDNA such as surface-enhanced Raman scattering (SERS) and biosensors are being studied, which may compete and replace traditional techniques (polymerase chain reaction and NGS) (129–133).

## Treatment Updates

### Current Management of Biliary Tract Cancers

Gemcitabine/cisplatin (GC) combination is the systemic therapy of choice in advanced BTC, and capecitabine is the recommended adjuvant treatment (in case of negative margins) (134, 135). The NCCN guidelines recommend FOLFOX based on the ABC-06 trial, and the difference in OS was minimal even though statistically significant (6.2 months in the FOLFOX group *vs.* 5.3 months in the supportive therapy group) (136). GC and the nab-paclitaxel combination seem promising, especially in the first line (49, 137). In a recently presented trial, nanoliposomal irinotecan (nal-IRI) plus 5-FU/LV was compared with 5-FU/LV in a phase IIb randomized trial (138). The PFS (7.1 months for the combination *vs.* 1.4 months, *p* = 0.019), OS (8.6 *vs.* 5.5 months, *p* > 0.001), and ORR (14% *vs.* 5.8%) were better for the combination therapy which is encouraging. The serious adverse events (≥ grade 3) were very high in the combination group (77% *vs.* 31%).

### Targeted Therapy

In FGFR2 fusions or rearrangements, pemigatinib and infigratinib are recommended (139). IDH1 or IDH1 mutant BTCs can be treated with ivosidenib (14). Neurotrophic tyrosine receptor kinase (NTRK) gene rearrangement-positive tumors respond to NTRK inhibitors such as larotrectinib and entrectinib (10, 11). Dabrafenib and trametinib combination was recently approved for *BRAF V600E* mutation (140, 141). The list of targeted therapies is summarized in **Table 4**. The published trials are cited, and the identifier is added to unpublished trials.

**Table 4:** Targeted therapy in biliary tract cancer.

Target	Drug	Approved	Early success	Ongoing
FGFR2	Pemigatinib	Pemigatinib**		Pemigatinib + GC (NCT03656536)^III^ *
	Infigratinib	Infigratinib**		Infigratinib *vs.* gemcitabine cisplatin (NCT03773302)^III^ *
	Futibatinib		Futibatinib** ^II^ (142)	Futibatinib *vs.* GC (NCT04093362)* ^III^
	Derazantinib		Derazantinib** ^I/II^ (143)	Ponatinib (NCT02265341)** ^II^
	Erdafitinib		Erdafitinib** ^II^ (144)	Deibo 137 (NCT03834220)** ^II^
IDH1	Ivosidenib	Ivosidenib**		
	LY3410738 (for IDH1 R132)			LY3410738 (NCT04521686)** ^I^
	Olutasidenib (FT-2102)			FT-2102 *vs.* FT-2102 and nivolumab (NCT03684811)** ^I/II^
IDH1/2	Dasatinib			Dasatinib (NCT02428855)** ^II^
	AG-881			AG-881 (NCT02481154)** ^I^
IDH2				Enasidenib (NCT02273739)** ^I/II^
NTRK	Larotectinib	Larotectinib**		
	Entrectinib	Entrectinib**		
Protein kinase CK2 inhibitor	Silmitasertib		Silmitasertib + GC* ^I/II^ (145)	
VEGF inhibitor	Regorafenib		Regorafenib** ^I^ (146)	Regorafenib (NCT02053376)** ^II^
	Surufatinib		Surufatinib** ^II^ (147)	
HER2 inhibitor	Zanidatamab		Zanidatamab** ^I/II^ (148, 149)	Zanidatamab (NCT04466891)** ^II^
	Neratinib		Neratinib** ^II^ (150)	
	Trastuzumab			Trastuzumab + pertuzumab** ^II^ (151)
				Trastuzumab + mFOLFOX** ^II^ (152)
Dickkopf-1 (DKK1)	DKN-01		DKN-01 + GC* ^I^ (128)	DKN-01 + N (NCT04057365)** ^II^
ATR inhibitor	Ceralasertib (Ce) (AZD6738)			Ce + D (NCT03780608)** ^II^
				Ce + olaparib (NCT03878095)** ^II^
BRCA1/2 inhibitor	Olaparib			Olaparib (NCT04042831)* ^II^
	Niraparib			Niraparib (NCT03207347)* ^II^
CDK4/6	Abemaciclib			Abemaciclib (NCT04003896)** ^II^
HDAC	Entinostat			Entinostat + nivolumab (NCT03250273)** ^II^

I, phase 1 trials; II, phase 2 trials; III, phase 3 trials; *first line; **second line or more; NTRK, neurotrophic tyrosine receptor kinase; ATR, ataxia telangiectasia and Rad3-related; HDAC, histone deacetylase; BRCA, breast cancer gene; VEGF, vascular endothelial growth factor; HER2, human epidermal growth factor receptor 2; IDH, isocitrate dehydrogenase; FGFR, fibroblast growth factor receptor.

### Immunotherapy

Treating disease by modulating (suppressing or activating) the immune system refers to immunotherapy (153). ICI is the most commonly used for immunotherapy in oncology and is used in almost all solid and hematological malignancies. In clinical practice, after progression in GC, targeted therapy or immunotherapy is considered if feasible. In patients with microsatellite instability (MSI-H), pembrolizumab is recommended based on the KEYNOTE-158 study with 22 advanced BTCs. The objective response rate was observed in 41% of BTCs with two patients with complete response (154). Correlation between TMB and response to ICI is difficult to assess as none of the BTCs enrolled in KEYNOTE-158 had higher TMB (≥10 mutations/Mb). PDL-1 overexpression does not seem to have any effect on responses either (155, 156). Nivolumab and ipilimumab combination did not have encouraging results either, with just 23% ORR (157). The current NCCN recommendation is to use nivolumab for refractory advance BTCs that are not MSI-H irrespective of TMB, and PDL-1 expression is weak (156).

Currently, many trials are combining ICI with either chemotherapy or targeted therapy or locoregional therapies such as transarterial chemoembolization (TACE), cryotherapy, radiofrequency ablation (RFA), and radiotherapy (as reported in **Table 5**). Traditional ICIs target either PD-1 or PD-L1 or CTLA-4. Bintrafusp alfa, a bifunctional antibody targeting TGF-β (transforming growth factor-β) and PD-L1, had success in the early phase trials (162). Vaccines with individual peptides such as MUC1, Wilms tumor 1 (WT1), or multiple in treating advanced BTC peptides had limited success previously but not enough to pursue it forward (170–175). There is some evidence that chimeric antigen receptor-modified T cells (CART) against epidermal growth factor receptor (EGFR) and CD133 and tumor-infiltrating lymphocytes are effective in managing refractory CCA (176–178).

**Table 5:** Immunotherapy in biliary tract cancers.

Drug class	Drug	Approved	Early success	Ongoing trials
Immune checkpoint inhibitor	Pembrolizumab (P)	P** in MSI-H	P + lenvatinib** ^II^ (158)	P + GC *vs.* placebo + GC* ^III^ (159)
				P + sargramostim (NCT02703714)** ^II^
				P + Olaparib (NCT04306367)** ^II^
				XmAb^®^22841 monotherapy *vs.* XmAb^®^22841 + P (NCT03849469)** ^I^
				P + XL888 (Hsp90 inhibitor)** ^I^ (NCT03095781)
				P + Peginterferon alpha-2b** ^II^ (NCT02982720)
	Nivolumab (N)	N**	N + GC *vs.* N + ipilimumab* ^II^ (160)	N + S-1+ G (NCT04172402)* ^II^
			N + GC** ^I^ (161)	N + rucaparib (NCT03639935)* ^II^
				Nal-irinotecan/5-fluorouracil/leucovorin + N (NCT03785873)** ^I/II^
				N + high-dose XRT *vs.* N + ipilimumab and high-dose XRT (NCT02866383)** ^II^
				N + TPST-1120 (PPARα antagonist)* ^I^ (NCT03829436)
	Bintrafusp alfa		Bintrafusp alfa** ^II^ (162)	Bintrafusp alfa + GC* ^II/III^ (163)
	Durvalumab (D) ± tremelimumab (T)		D ± T + GC (164)	D + GC (NCT03875235)* ^III^
			D and D + T** ^I^ (165)	D + olaparib (NCT03991832)** ^II^
				D + AZD6738 (ATP inhibitor) + olaparib (NCT04298021)** ^II^
				D *vs.* D + T (NCT04238637)** ^II^
				D + guadecitabine (NCT03257761)** ^I^
			T + TACE or RFA** ^II^ (166)	D + T *vs.* D + T + TACE *vs.* D + T + RFA *vs.* D + T + cryotherapy (NCT02821754)** ^II^
				D + SNDX-6352 (NCT04301778)** ^II^
				D + T + XRT (NCT03482102)** ^II^
	Toripalimab (To)		To + GC* (167)	
			To + lenvatinib + GEMOX (168)	To + GEMOX (NCT04191343)* ^II^
				To (IV) + HAIC infusion of oxaliplatin, 5-FU, and Bev (NCT04217954)* ^II^
	Camrelizumab (C)			C + cryoablation (NCT04299581)** ^II^
				C + radiotherapy (NCT03898895)* ^II^
	Sintlimab		Sintlimab (PD-1) + anlotinib (AL3818) (169)	
	STI-3031			STI-3031 (NCT03999658)** ^II^
	SHR-1210			SHR-1210 + capecitabine (NCT04295317)** ^II^
	Avelumab			Avelumab + regorafenib (NCT03475953)** ^I/II^
Arginase inhibition	NCB001158			NCB001158 + GC* ^I/II^ (13)
Natural killer (NK) cells	Allogenic NK cell			Allogenic NK cell (SMTNK) + P (NCT03937895)** ^I/I^
Autologous cells	T cells			Tumor-infiltrating lymphocytes with high-dose aldesleukin** ^II^ (NCT03801083)
				Central memory T cells + standard therapy after resection* ^II^ (NCT03820310)
CAR-T cell therapy	CAR-T			MUC1 CAR-T cell therapy + fludarabine + cyclophosphamide* ^I/II^ (NCT03633773)
				Anti-HER2 CAR-T cell* ^I^ (NCT04660929)
Oncolytic virus	Adenovirus			Virus encoding immunostimulatory TMZ-CD40L and 4-1BBL with GC* ^I/II^ (NCT03225989)

I, phase 1 trials; II, phase 2 trials; III, phase 3 trials; *, first line; **, second line or more; GC, gemcitabine/cisplatin; CAR-T, chimeric antigen receptor—T cell; MSI-H, microsatellite instability—high.

## Conclusion

BTCs are rare cancers with a high mortality rate and limited systemic options. It is important to recognize the significant differences in the genomic landscape of IHC, EHC, and GBC. As noted above, we need to start investing in DNA markers other than somatic mutations such as methylation markers and non-DNA markers (miRNA, mRNA, and lncRNA) to help diagnose, screen, and predict the treatment response in BTCs. There is also a critical need to explore and refine biomarkers (DNA and non-DNA) in blood, bile, and cytology specimens, as they are more accessible than tissue in BTCs. The low prevalence of MSI-H and targetable mutations in BTC restricts the use of available/approved therapies. The success of drugs targeting new targets (such as *PKCK2*, *HER2*, and *DKK1*) and newer drugs for older targets (FGFR inhibitors such as futibatinib, derazantinib, and erdafitinib) is encouraging, and we may have a host of new drugs in the next 3–4 years. Traditional PD-1, PD-L1, and CTLA-4 inhibitors are being tested in various ways, as monotherapy, in combination with targeted therapy or chemotherapy, or locoregional therapies for treating BTCs, which might open up a whole new arsenal of drugs to choose. Newer immunotherapies such as bintrafusp alfa, arginase inhibitors, and T-cell-mediated treatments can further expand the horizon in the management of BTCs.

## Author Contributions

Conceptualization: AsM. Writing—original draft preparation: AsM. Writing—review and editing: ArM, EW, and AT. All authors contributed to the article and approved the submitted version.

## Conflict of Interest

The authors declare that the research was conducted in the absence of any commercial or financial relationships that could be construed as a potential conflict of interest.

## Publisher’s Note

All claims expressed in this article are solely those of the authors and do not necessarily represent those of their affiliated organizations, or those of the publisher, the editors and the reviewers. Any product that may be evaluated in this article, or claim that may be made by its manufacturer, is not guaranteed or endorsed by the publisher.
